# Revisiting the intraperoxisomal pathway of mammalian PEX7

**DOI:** 10.1038/srep11806

**Published:** 2015-07-03

**Authors:** Tony A. Rodrigues, Cláudia P. Grou, Jorge E. Azevedo

**Affiliations:** 1 Instituto de Investigação e Inovação em Saúde, Universidade do Porto, Portugal; 2 Organelle Biogenesis and Function Group, Instituto de Biologia Molecular e Celular (IBMC), Universidade do Porto, Rua do Campo Alegre 823, 4150-180 Porto, Portugal; 3 Instituto de Ciências Biomédicas Abel Salazar (ICBAS), Universidade do Porto, Rua de Jorge Viterbo Ferreira 228, 4050-313 Porto, Portugal

## Abstract

Newly synthesized peroxisomal proteins containing a cleavable type 2 targeting signal (PTS2) are transported to the peroxisome by a cytosolic PEX5-PEX7 complex. There, the trimeric complex becomes inserted into the peroxisomal membrane docking/translocation machinery (DTM), a step that leads to the translocation of the cargo into the organelle matrix. Previous work suggests that PEX5 is retained at the DTM during all the steps occurring at the peroxisome but whether the same applies to PEX7 was unknown. By subjecting different pre-assembled trimeric PEX5-PEX7-PTS2 complexes to *in vitro* co-import/export assays we found that the export competence of peroxisomal PEX7 is largely determined by the PEX5 molecule that transported it to the peroxisome. This finding suggests that PEX7 is also retained at the DTM during the peroxisomal steps and implies that cargo proteins are released into the organelle matrix by DTM-embedded PEX7. The release step does not depend on PTS2 cleavage. Rather, our data suggest that insertion of the trimeric PEX5-PEX7-PTS2 protein complex into the DTM is probably accompanied by conformational alterations in PEX5 to allow release of the PTS2 protein into the organelle matrix.

Peroxisomes are single membrane-bound organelles present in almost all eukaryotic organisms. In mammals they are involved in many metabolic pathways such as beta-oxidation of fatty acids, synthesis of ether glycerophospholipids and bile acids, catabolism of some amino acids, and detoxification of hydrogen peroxide (reviewed in Ref. 1). The importance of these organelles in human health is dramatically illustrated by a group of genetic diseases, the so-called peroxisomal biogenesis disorders, in which peroxisomes are partially or completely dysfunctional^2^,^3^. In most cases, the genes affected in these devastating disorders encode proteins mechanistically involved in sorting newly synthesized proteins to the matrix of the organelle^4^,^5^.

Correct sorting of peroxisomal matrix proteins requires, on one hand, the existence of peroxisomal targeting signals (PTS) in their polypeptide chains, and on the other, a machinery that recognizes them and promotes their translocation across the organelle membrane. There are two types of PTSs. The PTS type 1 (PTS1) is present in the majority of peroxisomal matrix proteins and consists of a small peptide in their carboxy-terminus generally ending with the sequence S-K-L^6^,^7^. The other signal, the PTS2, is found in only a small number of proteins. It is a degenerated nonapeptide located at the N-termini of those proteins^8^,^9^ which in mammals and plants is cleaved by the peroxisomal matrix peptidase TYSND1/DEG15^10^,^11^,^12^. PTS1 proteins are recognized and transported to the peroxisome by the shuttling receptor PEX5 in all organisms characterized up to now^13^,^14^,^15^,^16^,^17^,^18^, whereas PTS2 proteins are recognized by a cytosolic PEX5-PEX7 protein complex in almost all organisms^19^,^20^,^21^,^22^,^23^. The exceptions to this rule are yeasts and fungi which rely on a complex comprising PEX7 and a species-specific protein (i.e., PEX20 or PEX18 or PEX21) to sort their PTS2 proteins to the peroxisome^24^,^25^,^26^,^27^.

According to current models, after binding PTS1 or PTS2 proteins in the cytosol the receptors PEX5 or PEX5-PEX7 interact with the peroxisomal docking/translocation machinery (DTM) (reviewed in Refs 28, 29, 30, 31). This is a transmembrane multisubunit protein complex comprising PEX13, PEX14 and the RING finger peroxins PEX2, PEX10 and PEX12^32^,^33^. After an initial docking step, the receptor-cargo complexes are inserted into the DTM with the concomitant translocation of the cargo proteins into the organelle lumen^34^,^35^,^36^. At this stage, PEX5 presents a transmembrane topology exposing a short N-terminal domain into the cytosol, whereas the bulky part of its polypeptide chain faces the peroxisomal matrix^37^,^38^. The data for PEX7 are not so clear. This protein may either be retained at the DTM, or completely translocated into the organelle matrix, presumably together with the PTS2 protein it transports^39^,^40^ (see also below). Remarkably, no cytosolic NTPs are necessary up to this stage of the peroxisomal protein import pathway^34^,^35^,^40^,^41^. This suggests that the driving force for cargo translocation into the peroxisomal matrix derives from the strong protein-protein interactions that are established between the receptor on one hand, and components of the DTM on the other^42^,^43^,^44^,^45^. Following the cargo translocation step, the peroxisomal import machinery has to be reset, i.e., the receptors have to be recycled back into the cytosol in order to promote additional cycles of protein transportation. The resetting process is well characterized for PEX5 and involves three steps. First, PEX5 is monoubiquitinated at a conserved cysteine residue (cysteine 11 in the human protein)^46^,^47^. This marks the DTM-embedded receptor for the second step, the ATP-dependent extraction back into the cytosol by the so-called receptor export module (REM), a protein complex comprising the two ATPases PEX1 and PEX6 plus PEX26, their peroxisomal membrane anchor^41^,^46^,^48^,^49^. Finally, the thioester-linked ubiquitin is removed from PEX5 probably by a combination of enzymatic and non-enzymatic events^50^,^51^,^52^.

As stated above, it is presently unknown whether PEX7 is completely translocated across the peroxisomal membrane during the protein transport process or if it is retained at the DTM during all the steps occurring at the peroxisome. In the absence of this information, simple but important mechanistic questions regarding the peroxisomal protein import machinery remain unanswered. One such question regards the cargo release step: where and how does PEX7 release its PTS2 cargoes ? Another one regards the properties of the DTM itself. For instance, if it turns out that PEX7 is completely translocated into the organelle lumen then we would have to conclude that the DTM functions as a bidirectional protein translocase every time a PTS2 protein is imported because for every PEX7-PTS2 complex translocated into the organelle matrix, a PEX7 molecule would have to be exported into the cytosol. In this work we describe several experiments that address these questions.

## Results and Discussion

### The export competence of peroxisomal PEX7 is determined by the PEX5 molecule that transported it to the organelle

We have recently optimized a rat liver post-nuclear supernatant (PNS)-based *in vitro* system to study the peroxisomal import/export mechanism of PEX7^40^. In brief, this system consists in incubating ^35^S-labeled PEX7 with a rat liver PNS fortified with recombinant pre-phytanoyl-CoA hydroxylase (prePHYH), a PTS2-containing protein, and recombinant ΔC1PEX5L, a protein comprising amino acids 1–324 of the so-called large isoform of PEX5^19^,^22^. ΔC1PEX5L possesses an intact PEX7-binding domain as well as all the domains necessary for a productive interaction with the peroxisomal DTM and, accordingly, it is still functional in the PTS2-mediated protein import pathway^19^,^22^,^34^,^40^. However, because it lacks the C-terminal half of PEX5 it cannot bind efficiently PTS1 proteins. This property minimizes competition phenomena involving endogenous (and abundant) PTS1 proteins that are always present in rat liver PNS and radiolabeled PEX7/PTS2 proteins used in these assays (see also Ref. 34). At the end of the incubation, the reactions are treated with a large amount of a protease to degrade non-imported (accessible) radiolabeled protein and the organelles are sedimented and analyzed by SDS-PAGE/autoradiography. This *in vitro* system can also be used to study the export mechanism of peroxisomal PEX7. In this case, after incubation to allow import of the radiolabeled protein, further import is blocked either by addition of a vast excess of recombinant NDPEX14 to the assay (NDPEX14 binds, and therefore neutralizes soluble PEX5^35^,^40^) or by sedimenting and resuspending the organelles in import buffer lacking radiolabeled protein; the organelles are then subjected to a second incubation to promote export of the shuttling receptors back into the soluble fraction^40^. Finally, after protease treatment, the organelles are analyzed as described above.

Using this strategy we have shown that PEX7 engaged in the transport of prePHYH to the peroxisome transiently acquires a protease-protected status exposing at least its N-terminus into the organelle matrix^40^. Additionally, we found that export of peroxisomal PEX7 back into the cytosol occurs only when PEX5 is also exported. Indeed, when PEX7 was transported to the peroxisome by a mutant PEX5 species that cannot be mono-ubiquitinated because it lacks the conserved cysteine 11 (i.e., ΔC1PEX5L(C11A)), the subsequent export of PEX7 back into the cytosol was no longer possible. Importantly, despite this dependency, peroxisomal PEX5 and PEX7 were found to exit the organelle with different kinetics suggesting that the two processes are not strictly coupled. This observation together with the fact that a small fraction of peroxisomal PEX7 can be extracted from the organelle by mild sonication (note that this technique can also extract proteins weakly associated with membranes) hampered us from drawing conclusions regarding the intraperoxisomal pathway followed by PEX7. In other words, it was not possible to determine whether PEX7 is retained at the DTM during the steps occurring at the peroxisome, as PEX5 is, or if the protein is completely translocated across the organelle membrane together with the PTS2-cargo it transports.

Considering that each rat liver peroxisome contains hundreds of DTMs (see Methods), most of which are functional in our *in vitro* assays (see Fig. 1 in Ref. 41), we reasoned that it might be possible to address this issue by co-importing two PEX7 species, each of which was pre-incubated with either an export-competent or -incompetent PEX5 protein, and determine how their export capacity is affected by the pre-incubation step. (Note that both the export-competent and -incompetent PEX5 proteins (ΔC1PEX5L and ΔC1PEX5L(C11A), respectively) are equally active in promoting import and processing of a PTS2 protein at the peroxisomal matrix^34^,^40^; see also below). If PEX7 is retained at the DTM during its passage through the peroxisome, then the export capacity of a given PEX7 should be determined by the PEX5 protein with which it was pre-incubated. If, on the contrary, PEX7 is released together with its cargo into the matrix of the organelle then its subsequent export should be independent of the PEX5 species that transported it to the organelle because, once in the matrix, PEX7 would be able to exit the organelle through any DTMs that remained vacant or that were occupied by export-competent PEX5 species.

**Figure 1:**
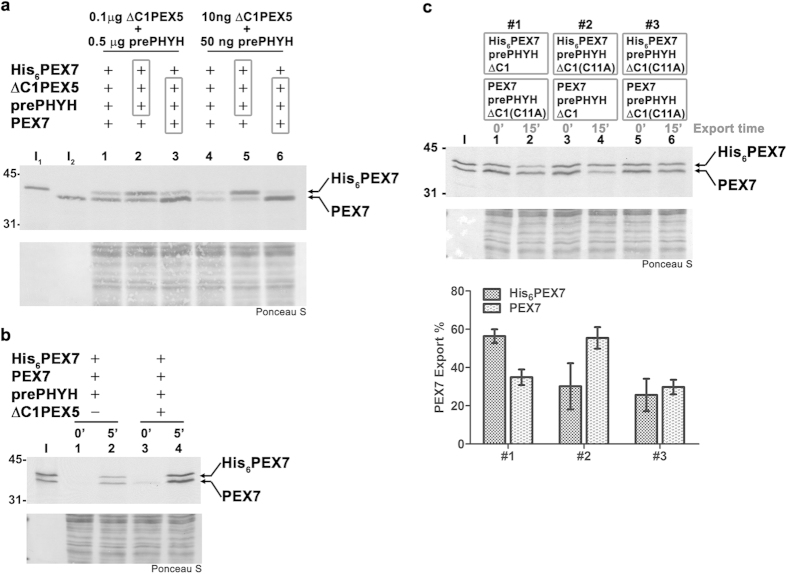
The export competence of peroxisomal PEX7 is determined by the PEX5 molecule that transported it to the organelle. **(a)** Two sets of three chemically identical co-import reactions of radiolabeled PEX7 and radiolabeled His-tagged PEX7 (His_6_PEX7) were performed with the indicated amounts of recombinant ΔC1PEX5L and prePHYH. Recombinant proteins were either added directly to the reaction (lanes 1 and 4) or pre-incubated with one of the radiolabeled PEX7 proteins, as indicated by boxes enclosing the “+” signs, before being added to the reaction containing the other PEX7 protein (lanes 2 and 5 or lanes 3 and 6 for His_6_PEX7 or PEX7, respectively). Import assays were made at 23 °C for 5 min to minimize receptor export. Pronase-treated organelles were subjected to SDS-PAGE/western-blot. The Ponceau S-stained membrane (lower panel) and its autoradiography (upper panel) are shown. Lanes I_1_ and I_2_, 2.5% of the reticulocyte lysates containing ^35^S-labeled His_6_PEX7 and PEX7 used in each reaction. **(b)** A mixture of radiolabeled His_6_PEX7 and PEX7 was pre-incubated with 50 ng of prePHYH in the presence or absence of ΔC1PEX5L (10 ng) and subjected to import assays. Aliquots were removed at 0 and 5 min and processed as above. **(c)** Reticulocyte lysates containing His_6_PEX7 or PEX7 were pre-incubated individually with 25 ng of prePHYH and 5 ng of either ΔC1PEX5L or ΔC1PEX5L(C11A) for 20 min at 23 °C. These mixtures were then added to a rat liver PNS in import buffer containing ATP in three different combinations (#1–#3), as indicated. After 5 min at 23 °C, import was inhibited by adding NDPEX14 (30 μM, final concentration) and the reactions were further incubated at 37 °C. Aliquots from each reaction were taken 0 and 15 min after adding NDPEX14. Pronase-treated organelles were analyzed as above. Lane I, 2.5% of the reticulocyte lysates containing radiolabeled PEX7 and His-tagged PEX7 were mixed and loaded together in the same lane. The bar graph shows averages and standard deviations (n = 4) of the amounts of PEX7 and His-tagged PEX7 exported in 15 min. Numbers to the left indicate the molecular masses of the protein standards in kDa.

Such co-import experiments should be feasible provided that: 1) the de novo formation of PEX5-PEX7-cargo complexes during the import assay is kept to a minimum, and 2) the half-life of the PEX5-PEX7-cargo interaction is relatively large so that PEX7 proteins pre-incubated with different recombinant PEX5 proteins do not exchange partners during the import assay. Figure 1a shows two sets of three chemically identical import reactions programmed with two radiolabeled versions of PEX7 that can be easily resolved by SDS-PAGE, His-tagged PEX7 and PEX7 (lanes I_1_ and I_2_, respectively). The first set of reactions (lanes 1–3) contained 500 ng of recombinant prePHYH and 100 ng ΔC1PEX5L (the amounts used in our previous work^40^) whereas in the second set (lanes 4–6) the amounts of both recombinant proteins were decreased 10-fold. The only difference between reactions in each set regards the way how radiolabeled PEX7 proteins and recombinant ΔC1PEX5L and prePHYH were handled before the import reactions. In one assay the proteins were added to import reactions individually (lanes 1 and 4), whereas in the other two reactions one of the two ^35^S-labeled PEX7 versions was pre-incubated with ΔC1PEX5L and prePHYH before starting the import reaction (lanes 2, 3, 5 and 6). As shown in Fig. 1a, the total amounts of imported PEX7 proteins in each of the three reactions containing standard amounts of ΔC1PEX5L and prePHYH (lanes 1–3) do not differ much. Nevertheless, it is already apparent that pre-incubating a given PEX7 protein with the recombinant proteins provides some kinetic advantage to that PEX7 species in the subsequent import assay (compare lane 1 with lanes 2 and 3), suggesting that the ΔC1PEX5L-PEX7-prePHYH interaction is indeed relatively stable (see also below). The kinetic advantage provided by the pre-incubation step becomes particularly evident in assays containing 10-fold less recombinant proteins (compare lane 4 with 5 and 6), probably because assembly of trimeric complexes during the import reaction is now a rate-limiting event. It is important to note that even under these conditions some radiolabeled PEX7 protein is still imported in a pre-incubation-independent manner (Fig. 1a, lane 4). Such import of ^35^S-PEX7 is probably due to endogenous rat liver PEX5, which is in fact more abundant than the recombinant PEX5 species added to these assays (there are approximately 50 ng of rat PEX5 per reaction; see Methods for details). Indeed, as shown in Fig. 1b, the import yield of radiolabeled PEX7 in assays containing recombinant prePHYH alone (50 ng) is still 25–30% of that obtained in the presence of both ΔC1PEX5L (10 ng) and prePHYH (compare lanes 2 and 4). Thus, any ^35^S-labeled PEX7 that does not associate with the recombinant PEX5 proteins during the pre-incubation step or, that having done so, dissociates from the recombinant proteins during the import assay, will form a complex with endogenous PEX5 and, subsequently, will be imported into and exported from peroxisomes.

We next performed import/export assays with the two ^35^S-labeled PEX7 proteins, each of which was individually pre-incubated with low amounts of recombinant prePHYH and either ΔC1PEX5L or export-incompetent ΔC1PEX5L(C11A). As shown in Fig. 1c, when both PEX7 proteins were pre-incubated with ΔC1PEX5L(C11A), approximately 30% of each radiolabeled protein was exported after a 15 min incubation (upper panel, lanes 5 and 6; see also lower panel for a quantification of the data). As explained above, the considerable basal export of PEX7 observed under these conditions is probably due to endogenous rat PEX5. Importantly, when ΔC1PEX5L was used in the pre-incubation step the amounts of exported ^35^S-labeled PEX7 proteins, be it His-tagged PEX7 (Fig. 1c, lanes 1 and 2) or PEX7 (lanes 3 and 4), were now almost two-fold larger. Apparently, the export competence of peroxisomal PEX7 is largely determined by the PEX5 protein with which it associated prior to import. Thus, these results strongly suggest that, similarly to PEX5, PEX7 engaged in the PTS2-import pathway is retained at the DTM during all the steps occurring at the peroxisome.

### Release of the PTS2 cargo protein by PEX7 at the DTM does not require cleavage of the PTS2

**Figure 2:**
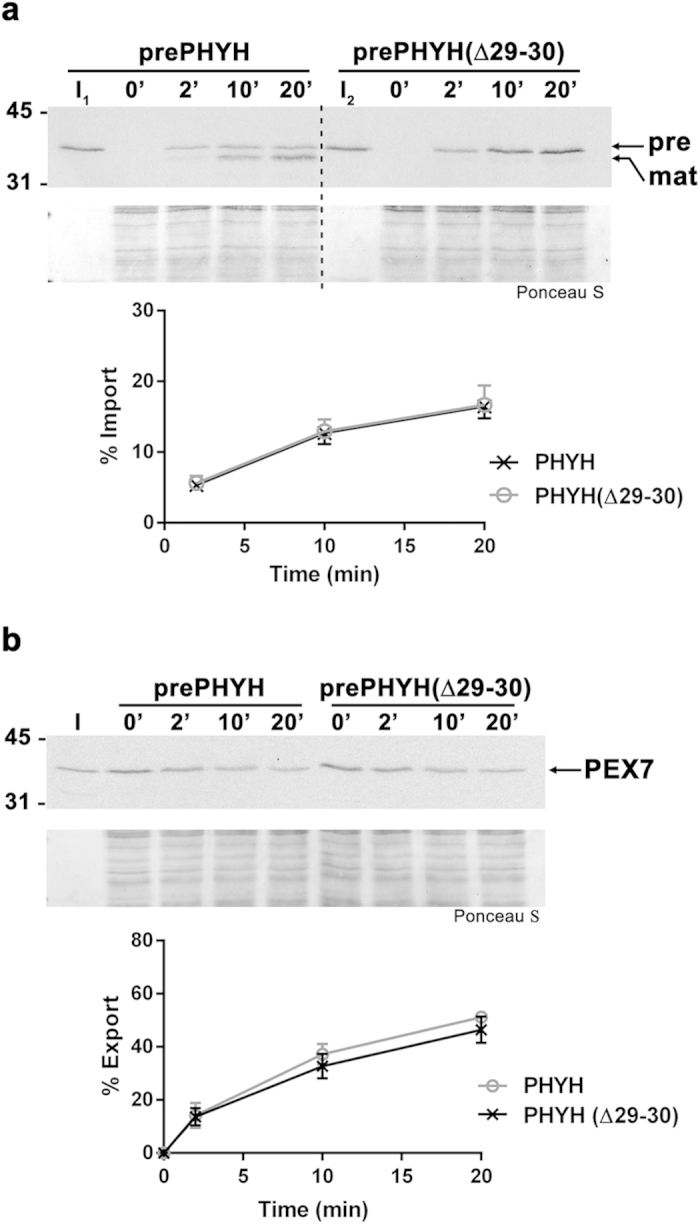
Proteolytic cleavage of the PTS2 peptide is not necessary for the cargo-release step. **(a)** PrePHYH and an non-cleavable form of it, prePHYH(Δ29-30), accumulate in peroxisomes at similar rates. A rat liver PNS was incubated with either radiolabeled prePHYH or prePHYH(Δ29-30) in import buffer containing ATP, recombinant ΔC1PEX5L and “cold” *in vitro* synthesized PEX7 at 37 °C. Aliquots were taken at the indicated time points. Pronase-treated organelles were analyzed by SDS-PAGE and blotted onto a nitrocellulose membrane. Lanes I_1_ and I_2_, 10% of the reticulocyte lysates containing radiolabeled prePHYH or prePHYH(Δ29-30) used in each reaction, respectively. Autoradiographs (upper panels) and corresponding Ponceau S-stained membranes (lower panels) are shown. “pre” and “mat” refer to the precursor and mature forms of PHYH. Numbers to the left indicate the molecular masses of the protein standards in kDa. The graph shows averages and standard deviations (n = 3) of the percentage of imported proteins at different time points. **(b)** PEX7 proteins that transported prePHYH or prePHYH(Δ29-30) to the peroxisome display similar export kinetics. Radiolabeled PEX7 was incubated with a rat liver PNS in import buffer containing ATP, recombinant ΔC1PEX5L and either recombinant prePHYH or prePHYH(Δ29-30) at 37 °C for 15 min. The organelles were then isolated by centrifugation, resuspended in fresh import buffer and further incubated at 37 °C in the presence of ATP to promote export of receptors. Aliquots were collected at the indicated time points. Pronase-treated organelles were analyzed as above. Lanes I, 5% of the reticulocyte lysate containing radiolabeled PEX7 used in each reaction. An autoradiograph (upper panel) and the corresponding Ponceau S-stained membrane (lower panel) are shown. Numbers to the left indicate the molecular masses of the protein standards in kDa. The graph shows averages and standard deviations (n = 3) of the percentage of exported PEX7 at different time points.

Upon arrival at the peroxisomal matrix, PTS2-containing proteins are processed by the matrix-localized protease TYSND1, thus losing their N-terminal PTS2 extensions. Although the exact timing of this cleaving step remains unknown, it was recently proposed that TYSND1 might act on the trimeric PEX5-PEX7-PTS2 cargo complex and thus actually trigger release of the cargo^53^. According to those authors, failure to cleave the PTS2 might even lead to a situation in which PTS2 proteins would not be released from the PEX5-PEX7 complex into the peroxisomal matrix and, instead, would exit the organelle together with the receptors. Aiming at better understanding how PTS2 proteins are released into the peroxisomal matrix, we decided to put this idea to the test. For this purpose we first synthesized a ^35^S-labeled version of prePHYH lacking amino acid residues 29-30 (a truncation that renders the protein uncleavable upon peroxisomal import; see below) and asked whether this protein (prePHYH(Δ29-30)) accumulates at the peroxisome as efficiently as the wild type protein in *in vitro* assays. The results presented in Fig. 2a show that the two proteins accumulate at the organelle at the same rate thus suggesting that no significant amount of imported uncleavable prePHYH(Δ29-30) protein was exported back into the cytosol during the time window of these *in vitro* assays. We still considered the possibility that imported prePHYH(Δ29-30) becomes stuck at the DTM because it cannot be released from the PEX5-PEX7 complex. If this were so, one would expect to find also PEX7 retained at the DTM, i.e., PEX7 export should also be affected. To test this hypothesis we subjected ^35^S-PEX7 to import assays in the presence of ΔC1PEX5L and either recombinant prePHYH or prePHYH(Δ29-30), and, after stopping further import by adding NDPEX14, we monitored the export rate of radiolabeled PEX7. As shown in Fig. 2b, the export rates of PEX7 in the two assays were essentially the same. We conclude that release of a PTS2 protein from PEX7 at the DTM does not require cleavage of the PTS2 by TYSND1.

### The half-life/affinity of the PEX7-PTS2 interaction is dramatically increased by PEX5 – implications for the PTS2 cargo release step

The results above suggest that release of a PTS2 protein into the peroxisomal matrix from DTM-embedded PEX7 is not triggered by processing of the signal peptide but provide no additional clues on how this step occurs. We do know from previous work that monoubiquitination of PEX5, although mandatory for the subsequent dislocation of PEX5 from the DTM by the ATP-dependent REM, is not required for cargo release^34^,^35^. Also, depletion of NTPs from import assays using apyrase does not block translocation into and processing of pre-thiolase in the peroxisomal matrix^34^. Seemingly, release of cargo proteins from DTM-embedded receptors does not require input of chemical energy. Two non-mutually exclusive mechanisms could be envisaged to explain how DTM-embedded receptors irreversibly release cargo proteins into the peroxisomal matrix. One possibility is that the strong protein interactions that are established between the receptor-cargo complex and the DTM during the insertion step are accompanied by conformational alterations of the receptors themselves that decrease or even abolish their capacity to bind cargoes. The other would be to assume that the DTM is a (flexible) trapdoor for cargo proteins allowing them to reach the matrix side of the peroxisomal membrane and be released in a spontaneous manner from the corresponding receptors, but blocking their subsequent interaction with the receptors.

**Figure 3:**
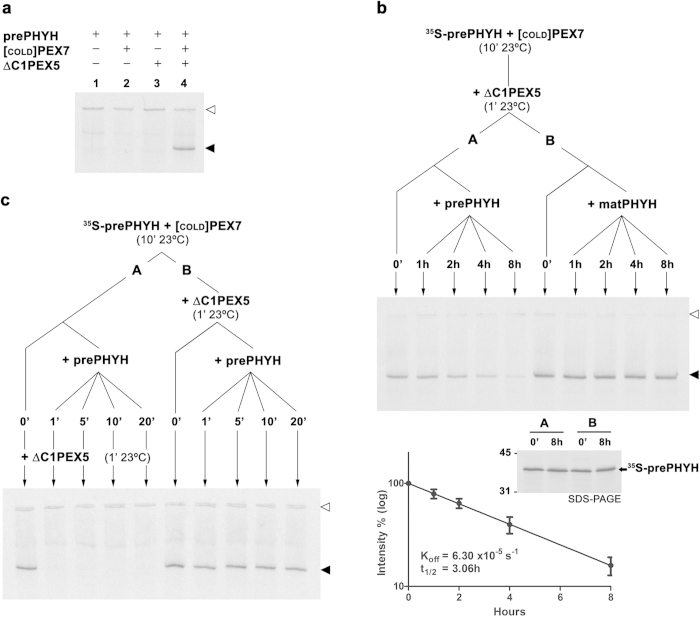
PEX5 dramatically increases the half-life/affinity of the PEX7-PTS2 interaction. (**a**) PrePHYH runs in native-PAGE only when mixed with ΔC1PEX5L and PEX7. Radiolabeled prePHYH was pre-incubated for 10 min at 23 °C in the absence or presence of *in vitro* synthesized “cold” PEX7, recombinant ΔC1PEX5L, or both, as indicated. Samples were analyzed by native-PAGE/autoradiography. (**b**) The residence time (half-life) of prePHYH in the trimeric ΔC1PEX5L-PEX7-prePHYH complex is very long. Radiolabeled prePHYH and “cold” PEX7 were incubated together for 10 min at 23 °C. Recombinant ΔC1PEX5L was then added and the mixture was incubated for 1 min to allow formation of the trimeric complex. The mixture was divided in two samples (tube A and B, respectively), and aliquots (t = 0′) were withdrawn and immediately flash-frozen in liquid N_2_. Recombinant prePHYH and mature PHYH (matPHYH) were then added to tubes A and B, respectively. Both tubes were incubated at 23 °C and aliquots were collected and frozen at the indicated time points. Samples were analyzed by native-PAGE (upper panel) and SDS-PAGE (middle panel). Autoradiographs are shown. The graph shows the logarithm of band intensities as function of time (averages and standard deviations are presented, n = 4; half-life = 3.06 h (95% confidence range of 2.23 h–4.90 h)). (**c**) The half-life/affinity of the PEX7-PTS2 interaction is very low. Radiolabeled prePHYH was incubated with PEX7 as above. The mixture was divided in two samples (tube A and B, respectively). One aliquot (t = 0′) was withdrawn from tube A, incubated with recombinant ΔC1PEX5L for 1 min and flash-frozen. Recombinant prePHYH was then added to tube A and aliquots were removed at the indicated time points, incubated with recombinant ΔC1PEX5L for 1 min and frozen as above. Tube B received recombinant ΔC1PEX5L and an aliquot (t = 0′) was removed after 1 minute and flash-frozen. Recombinant prePHYH was then added and aliquots were withdrawn and flash-frozen at the indicated time points. Samples were analyzed as above. The open (▷) and solid (▶) arrowheads indicate the wells of the native gel, and the trimeric complex containing ^35^S-prePHYH, respectively.

We reasoned that determining the half-life of the receptor-PTS2 cargo protein complex might shed some light on this issue. For instance, if this interaction turns out to be very stable, i.e., if its half-life is in the order of many minutes or even hours, then a simple trapdoor mechanism as the one described above would be rather improbable. For this purpose we incubated radiolabeled prePHYH with either PEX7 or both PEX7 and ΔC1PEX5L to allow formation of dimeric or trimeric complexes, respectively, added a vast excess of recombinant prePHYH to these mixtures, and removed aliquots at the different time points. Under these conditions any radiolabeled prePHYH that dissociates from the pre-assembled complexes during the time course analyses will not be able to reassociate with PEX7 or PEX7/ΔC1PEX5L because it will be out competed by the recombinant PTS2 protein. Samples were then analyzed by native-PAGE (see Methods for details). Note that prePHYH (theoretical pI of 8.7) runs in these native gels (pH 8.5) only when in complex with both PEX7 and ΔC1PEX5L (see Fig. 3a). Thus, in the experiments aiming at determining the half-life of the PEX7-prePHYH interaction, samples were incubated with ΔC1PEX5L for one minute before proceeding with the native-PAGE analyses. This short incubation with ΔC1PEX5L is sufficient to yield a very stable trimeric complex that, in a time window of 20 min or less, releases no significant amount of radiolabeled prePHYH (see below). The results of these experiments are shown in Fig. 3b,c. When a sample containing the pre-assembled trimeric complex ΔC1PEX5L-PEX7-^35^S-prePHYH was incubated with recombinant prePHYH, the amount of radiolabeled prePHYH in the complex decreased slowly over of period of hours, displaying a half-life of 3.06 h. SDS-PAGE analyses of aliquots withdrawn at the 0 and 8 h time points show that this decrease is not the result of proteolysis (Fig. 3b, middle panel). Accordingly, no such decrease was observed in a sample that received recombinant mature PHYH (i.e., a PHYH protein lacking the PTS2), indicating that the observed effect is specific. In contrast, incubation of a PEX7/^35^S-prePHYH mixture with recombinant prePHYH for just one minute was sufficient to completely block the subsequent formation of trimeric ΔC1PEX5L-PEX7-^35^S-prePHYH (see Fig. 3c). There are two possible interpretations for this result: 1) either the half-life of the PEX7-prePHYH interaction is extremely short (i.e., a few seconds at most), or 2) the K_d_ of this interaction is so large that no significant amount of PEX7-^35^S-prePHYH complex was actually present in the mixture before adding the recombinant proteins. In either case it is evident that in the absence of ΔC1PEX5L the PEX7-prePHYH interaction is rather weak/unstable. Together these results indicate that a putative trapdoor model involving the spontaneous release of the cargo protein from a DTM-embedded PEX5-PEX7-PTS2 protein complex is not possible. Clearly, some conformational alteration must occur in the receptor complex during its insertion into the DTM in order to disrupt the stabilizing effect of PEX5 on the receptor-cargo interaction.

Recently, Pan *et al*. reported the structure of a trimeric complex comprising yeast PEX7, an artificial PTS2 protein and a small fragment of PEX21, the yeast ortholog of mammalian/plant PEX5 in the PTS2-mediated protein import pathway^54^. That work revealed that the interaction between the PTS2 peptide and PEX7 is stabilized by the PEX21 peptide which interacts with both the PTS2 peptide and PEX7. Given the results above suggesting that the PEX7-PTS2 interaction is very weak/unstable in the absence of PEX5 we reasoned that the disruption of the PEX5-PTS2 interaction in the trimeric complex by some DTM component might be sufficient to trigger release of the cargo into the organelle matrix. An obvious candidate to perform this task would be PEX14, a central component of the DTM. Indeed, it has been reported that the N-terminal domain of PEX14 comprising its PEX5-interaction motif has the capacity to weaken or even abolish the interaction between PEX5 or the PEX5-PEX7 complex and their cargoes^43^,^44^,^55^. Thus, we asked whether addition of recombinant NDPEX14 to pre-assembled trimeric ΔC1PEX5L-PEX7-prePHYH complex would disrupt the receptor-cargo protein interaction. Despite several attempts we were unable to detect PEX14-induced disruption of the trimeric complex (not shown). Possibly, this in-solution binding assay does not recreate the avidity effects that presumably occur in the authentic situation when a cargo-loaded PEX5-PEX7 protein complex inserts into a membrane complex (i.e., the DTM) comprising several PEX14 molecules.

## Conclusion

**Figure 4:**
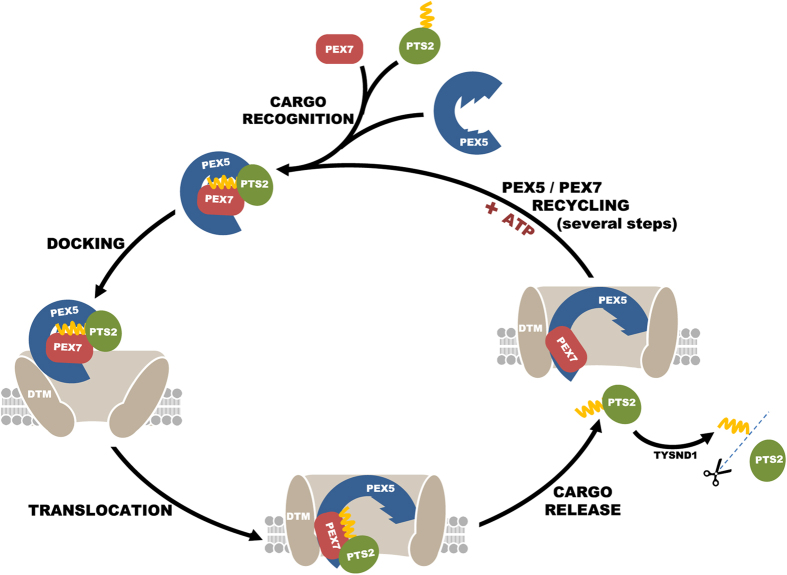
Working model for the PTS2-mediated protein import. PTS2-containing proteins are recognized by the cytosolic receptors PEX5 and PEX7 forming a highly stable trimeric complex. The cargo-receptor complex then interacts with the docking/translocation machinery (DTM) at the peroxisome membrane. The strong protein-protein interactions that occur at this stage result in the insertion of the cargo-receptor complex into the DTM. At this stage PEX5 displays a transmembrane topology, whereas PEX7 exposes a part of its polypeptide chain into the peroxisomal matrix. The insertion step also induces conformational alterations in PEX5, disrupting its strong stabilizing effect on the PEX7-PTS2 interaction, and thus triggering the release of the PTS2 protein into the peroxisomal matrix. Here, the PTS2-containing peptide is cleaved by TYSND1. After cargo release, the receptors are recycled back into the cytosol by an ATP-dependent machinery.

In mammals, plants and many other organisms PEX7 functions as an ancillary factor of PEX5 endowing this shuttling receptor the capacity to transport PTS2 proteins to the organelle. Previous work has shown that PEX7 enters the peroxisome compartment in a PEX5- and cargo-dependent manner, where it acquires a protease-resistant status, exposing at least its N-terminus into the organelle matrix^40^. However, whether mammalian PEX7 is completely translocated into the peroxisomal matrix together with its cargo, as proposed for the yeast protein^39^ or retained at the DTM during its journey through the organelle was unknown (see Ref. 40). In this work we provide evidence suggesting that mammalian PEX7, like PEX5, is retained at the DTM. Thus, similarly to PTS1 proteins, PTS2 cargoes are also released into the peroxisomal matrix from DTM-embedded receptors. Experiments aiming at understanding the mechanistic details of this event revealed that cleavage of the PTS2 by the matrix protease TYSND1 is not required for cargo release. On the other hand, we found that the PTS2-PEX7 interaction is quite weak/unstable but that binding of PEX5 to these two proteins yields a remarkably stable trimeric complex. Thus, in agreement with the structure of the yeast PEX21-PEX7-PTS2 complex and with very recent qualitative data on the human peroxins^56^, it seems that PEX5 acts as a locking device keeping the PTS2 protein tightly bound to PEX7. Given the efficiency with which PEX5 achieves this task (the half-life of prePHYH in the trimeric complex is approximately 3 h), we can only conclude that the interaction between PEX5 and the PTS2 protein in the trimeric PEX5-PEX7-PTS2 complex must be disrupted during insertion of the complex into the DTM. This then leaves the PTS2 cargo protein solely bound to PEX7, and thus presumably poised to be released into the peroxisomal matrix (see Fig. 4). Subsequent removal of the PTS2 peptide by the matrix processing peptidase would prevent reassociation of the cargo protein with PEX7, making the import process essentially irreversible. The irreversibility of the transport process might also be attained simply by rapidly dislocating PEX7 from DTM.

Our work does not provide clues on the identity of the DTM component that triggers the required conformational alteration on PEX5 leading to cargo release. Attempts to displace prePHYH from the trimeric PEX5-PEX7-prePHYH complex using recombinant NDPEX14 were unsuccessful. Nevertheless, given the limitations of the *in vitro* assay used here, and the fact that the PEX5 region corresponding to the yeast PEX21 domain that interacts with the PEX7-PTS2 complex contains PEX14-binding motifs, PEX14 is still a good candidate to perform this role. Clearly, improved *in vitro* protein-protein interaction assays that better mimic the *in vivo* situation are needed to confirm this hypothesis.

## Methods

### Plasmids

The plasmids encoding human PEX7 (pGEM4-PEX7), histidine-tagged PEX7 (pET28-PEX7) were recently described^40^. The cDNA encoding human prePHYH was obtained by PCR amplification of pQE31-prePHYH^57^ using the primers 5’-GACAAAGCTTGCCACCATGGAGCAGCTTCG-3’ and 5’-GGGCGCGAATTCTCAAAGATTGGTTCTTTCTC-3’ and cloned into the HindIII and EcoRI sites of pGEM4® (Promega) yielding pGEM4-prePHYH. Plasmids encoding a mutant version of Phytanoyl-CoA hydroxylase lacking residues Pro29 and Thr30, pQE31-prePHYH(∆29-30) and pGEM4-prePHYH(∆29-30), were obtained with the QuikChange® site-directed mutagenesis kit (Stratagene), using the primers 5’-GGGGCTGTCGTAGCTCATTCAGGGACTATTTCCTCTGC-3’ and 5’-GCAGAGGAAATAGTCCCTGAATGAGCTACGACAGCCCC-3’ and plasmids pQE31-prePHYH and pGEM4-prePHYH as the respective templates.

### *In Vitro* Synthesis of Proteins

Radiolabeled proteins were synthesized using the TNT^®^ T7 quick coupled transcription/translation kit (Promega) in the presence of [^35^S]methionine (specific activity >1000 Ci/mmol; PerkinElmer Life Sciences). Unlabeled (“cold”) PEX7 was synthesized using the same Kit but using the methionine provided in the Kit.

### Recombinant Proteins

The recombinant protein comprising amino acid residues 1–324 of the large isoform of human PEX5 (ΔC1PEX5L)^58^ and ∆C1PEX5L containing the missense mutation C11A (∆C1PEX5L(C11A))^40^, the precursor and mature forms of human Phytanoyl-CoA hydroxylase (prePHYH and matPHYH, respectively), mutant prePHYH(∆29-30)^57^, and a protein comprising the first 80 amino acid residues of human PEX14 (NDPEX14)^59^ were expressed and purified as described previously.

### *In Vitro* Import/Export Reactions

Rat liver post-nuclear supernatants (PNS) for *in vitro* assays were prepared exactly as described before^37^,^40^. In a typical import reaction (100 μl final volume), ^35^S-labeled proteins (1–2 μl of the rabbit reticulocyte lysates) were first diluted to 10 μl with import buffer (20 mM MOPS-KOH, pH 7.4, 0.25 M sucrose, 50 mM KCl, 3 mM MgCl_2_, 20 μM methionine, 2 μg/ml N-(trans-epoxysuccinyl)-L-leucine 4-guanidinobutylamide, 2 mM reduced glutathione, final concentrations) and added to 500 μg (protein) of PNS that had been primed for import (5 min incubation at 37 °C in import buffer containing 0.3 mM ATP; see^34^,^41^ for details). Unless otherwise stated, reactions were incubated at 37 °C. ATP was used at 3 mM, final concentration. In standard assays, the reactions also contained bovine ubiquitin (10 μM), recombinant ∆C1PEX5L or ∆C1PEX5L(C11A) (30 nM, final concentration), and recombinant prePHYH or p-PHYH(∆29-30) (140 nM, final concentration). Where indicated, the reticulocyte lysates containing ^35^S-PEX7 and ^35^S-His_6_PEX7 were pre-incubated with recombinant prePHYH and either ∆C1PEX5L or ∆C1PEX5L(C11A) for 20 min at 23 °C in 10 μl of import buffer. In the *in vitro* export assays, radiolabeled proteins were first subjected to an import incubation, and further import was then blocked either by adding recombinant NDPEX14 (30 μM, final concentration) or by isolating the organelles by centrifugation and resuspending them in import buffer. The organelle suspensions were then incubated at 37 °C in the presence of 5 mM ATP. Protease treatment of import/export assays and analyses by SDS-PAGE/autoradiography were done exactly as described recently^40^.

### Native-PAGE Analyses

The reticulocyte lysate containing radiolabeled prePHYH (0.25 μl) was incubated with 0.25 μl of a reticulocyte lysate containing unlabeled PEX7 (“cold” PEX7) for 10 min at 23 °C in 50 mM Tris-HCl, pH 8.0, 150 mM NaCl, 1 mM EDTA, 1 mM methionine, 1 mM DTT. Recombinant ∆C1PEX5L (1 μg), prePHYH (100 ng) or mature PHYH (100 ng) were added to the reticulocyte lysates as specified in figure legends. Samples were prepared for native-PAGE by adding 1 μl of a solution containing 0.17% (w/v) bromophenol blue, 50% (w/v) sucrose. The samples were loaded onto Tris nondenaturating 8% polyacrylamide gels using a discontinuous buffer system^60^ and run at 250 V at 4 °C for 2 h. The gels were blotted onto nitrocellulose membranes, stained with Ponceau S, and subjected to autoradiography.

### Miscellaneous

Wistar rats were handled in accordance with the protocols approved by the IBMC Animal Ethics Committee (CEA – Comissão de Ética Animal). Experimental protocols were approved by the Portuguese General Veterinarian Board (DGAV – Direcção Geral de Alimentação e Veterinária). Densitometric analyses of autoradiographs were performed using the ImageJ software (Rasband, W.S., ImageJ, U. S. National Institutes of Health, Bethesda, Maryland, USA, http://imagej.nih.gov/ij/,1997–2011). Plots showing averages and standard deviation (n = x, where x is the number of independent experiments) were done using GraphPad Prism version 6.00 for Windows, GraphPad Software, La Jolla California USA. The number or DTMs per peroxisomes was estimated from the following data. One gram of rat liver contains 260 mg of total protein of which 2.5% (6.5 mg) are peroxisomal proteins^61^,^62^. The number of peroxisomes/g of liver^61^,^62^ is 5.8 × 10^10^. PEX14, a 41-kDa protein, comprises approximately 0.25% of all peroxisomal protein^33^. Thus, there are 16.25 μg of PEX14 or 2.4 × 10^14^ molecules/g of liver. Dividing by the number of peroxisomes, this gives approximately 4,000 molecules of PEX14/peroxisome or, assuming that each DTM contains 7 molecules of PEX14^33^, 570 DTMs/peroxisome. The amount of endogenous rat liver PEX5 in the *in vitro* assays (500 μg of PNS/reaction) was estimated assuming that peroxisomes comprise 2.5% of PNS protein^61^ and that the PNS contains 4 ng of PEX5 per microgram of total peroxisomal protein^43^.

## Additional Information

**How to cite this article**: Rodrigues, T. A. *et al*. Revisiting the intraperoxisomal pathway of mammalian PEX7. *Sci. Rep*. **5**, 11806; doi: 10.1038/srep11806 (2015).

## References

[b1] Wanders R. J. Metabolic functions of peroxisomes in health and disease. Biochimie 98, 36–44 (2014). 24012550 10.1016/j.biochi.2013.08.022

[b2] Aubourg P. & Wanders R. Peroxisomal disorders. Hand. Clin. Neur. 113, 1593–1609 (2013). 10.1016/B978-0-444-59565-2.00028-9 23622381

[b3] Braverman N. E., D’Agostino M. D. & Maclean G. E. Peroxisome biogenesis disorders: Biological, clinical and pathophysiological perspectives. Dev. Dis. Res. Rev. 17, 187–196 (2013). 10.1002/ddrr.1113 23798008

[b4] Nagotu S., Kalel V. C., Erdmann R. & Platta H. W. Molecular basis of peroxisomal biogenesis disorders caused by defects in peroxisomal matrix protein import. Biochim. Biophys. Acta 1822, 1326–1336 (2012). 22617146 10.1016/j.bbadis.2012.05.010

[b5] Waterham H. R. & Ebberink M. S. Genetics and molecular basis of human peroxisome biogenesis disorders. Biochim. Biophys. Acta 1822, 1430–1441 (2012). 22871920 10.1016/j.bbadis.2012.04.006

[b6] Brocard C. & Hartig A. Peroxisome targeting signal 1: is it really a simple tripeptide? Biochim. Biophys. Acta 1763, 1565–1573 (2006). 17007944 10.1016/j.bbamcr.2006.08.022

[b7] Gould S. J., Keller G. A., Hosken N., Wilkinson J. & Subramani S. A conserved tripeptide sorts proteins to peroxisomes. J. Cell Biol. 108, 1657–1664 (1989). 2654139 10.1083/jcb.108.5.1657 PMC2115556

[b8] Lazarow P. B. The import receptor Pex7p and the PTS2 targeting sequence. Biochim. Biophys. Acta 1763, 1599–1604 (2006). 16996627 10.1016/j.bbamcr.2006.08.011

[b9] Swinkels B. W., Gould S. J., Bodnar A. G., Rachubinski R. A. & Subramani S. A novel, cleavable peroxisomal targeting signal at the amino-terminus of the rat 3-ketoacyl-CoA thiolase. EMBO J. 10, 3255–3262 (1991). 1680677 10.1002/j.1460-2075.1991.tb04889.x PMC453050

[b10] Helm M. . Dual specificities of the glyoxysomal/peroxisomal processing protease Deg15 in higher plants. Proc. Natl. Acad. Sci. USA 104, 11501–11506 (2007). 17592111 10.1073/pnas.0704733104 PMC2040927

[b11] Kurochkin I. V. . Novel peroxisomal protease Tysnd1 processes PTS1- and PTS2-containing enzymes involved in beta-oxidation of fatty acids. EMBO J. 26, 835–845 (2007). 17255948 10.1038/sj.emboj.7601525 PMC1794383

[b12] Schuhmann H., Huesgen P. F., Gietl C. & Adamska I. The DEG15 serine protease cleaves peroxisomal targeting signal 2-containing proteins in Arabidopsis. Plant Physiol. 148, 1847–1856 (2008). 18952862 10.1104/pp.108.125377 PMC2593680

[b13] Dodt G. . Mutations in the PTS1 receptor gene, PXR1, define complementation group 2 of the peroxisome biogenesis disorders. Nat. Genet. 9 (1995). 10.1038/ng0295-115 7719337

[b14] Jardim A., Liu W., Zheleznova E. & Ullman B. Peroxisomal targeting signal-1 receptor protein PEX5 from Leishmania donovani. Molecular, biochemical, and immunocytochemical characterization. J. Biol. Chem. 275, 13637–13644 (2000). 10788481 10.1074/jbc.275.18.13637

[b15] Kragler F., Lametschwandtner G., Christmann J., Hartig A. & Harada J. J. Identification and analysis of the plant peroxisomal targeting signal 1 receptor NtPEX5. Proc. Natl. Acad. Sci. USA 95, 13336–13341 (1998). 9789089 10.1073/pnas.95.22.13336 PMC23804

[b16] McCollum D., Monosov E. & Subramani S. The pas8 mutant of Pichia pastoris exhibits the peroxisomal protein import deficiencies of Zellweger syndrome cells–the PAS8 protein binds to the COOH-terminal tripeptide peroxisomal targeting signal, and is a member of the TPR protein family. J. Cell Biol. 121, 761–774 (1993). 8098333 10.1083/jcb.121.4.761 PMC2119792

[b17] van der Klei I. J. . The Hansenula polymorpha PER3 gene is essential for the import of PTS1 proteins into the peroxisomal matrix. J. Biol. Chem. 270, 17229–17236 (1995). 7615522 10.1074/jbc.270.29.17229

[b18] Van der Leij I., Franse M. M., Elgersma Y., Distel B. & Tabak H. F. PAS10 is a tetratricopeptide-repeat protein that is essential for the import of most matrix proteins into peroxisomes of Saccharomyces cerevisiae. Proc. Natl. Acad. Sci. USA 90, 11782–11786 (1993). 8265627 10.1073/pnas.90.24.11782 PMC48068

[b19] Braverman N., Dodt G., Gould S. J. & Valle D. An isoform of pex5p, the human PTS1 receptor, is required for the import of PTS2 proteins into peroxisomes. Hum. Mol. Genet. 7, 1195–1205 (1998). 9668159 10.1093/hmg/7.8.1195

[b20] Galland N. . Characterization of the role of the receptors PEX5 and PEX7 in the import of proteins into glycosomes of Trypanosoma brucei. Biochim. Biophys. Acta 1773, 521–535 (2007). 17320990 10.1016/j.bbamcr.2007.01.006

[b21] Mukai S. & Fujiki Y. Molecular mechanisms of import of peroxisome-targeting signal type 2 (PTS2) proteins by PTS2 receptor Pex7p and PTS1 receptor Pex5pL. J. Biol. Chem. 281, 37311–37320 (2006). 17040904 10.1074/jbc.M607178200

[b22] Otera H. . Peroxisome targeting signal type 1 (PTS1) receptor is involved in import of both PTS1 and PTS2: studies with PEX5-defective CHO cell mutants. Mol. Cell. Biol. 18, 388–399 (1998). 9418886 10.1128/mcb.18.1.388 PMC121509

[b23] Woodward A. W. & Bartel B. The Arabidopsis peroxisomal targeting signal type 2 receptor PEX7 is necessary for peroxisome function and dependent on PEX5. Mol. Biol. Cell 16, 573–583 (2005). 15548601 10.1091/mbc.E04-05-0422 PMC545895

[b24] Leon S. . Dynamics of the peroxisomal import cycle of PpPex20p: ubiquitin-dependent localization and regulation. J. Cell Biol. 172, 67–78 (2006). 16390998 10.1083/jcb.200508096 PMC2063535

[b25] Otzen M., Wang D., Lunenborg M. G. & van der Klei I. J. Hansenula polymorpha Pex20p is an oligomer that binds the peroxisomal targeting signal 2 (PTS2). J. Cell Sci. 118, 3409–3418 (2005). 16079284 10.1242/jcs.02463

[b26] Purdue P. E., Yang X. & Lazarow P. B. Pex18p and Pex21p, a novel pair of related peroxins essential for peroxisomal targeting by the PTS2 pathway. J. Cell Biol. 143, 1859–1869 (1998). 9864360 10.1083/jcb.143.7.1859 PMC2175223

[b27] Sichting M., Schell-Steven A., Prokisch H., Erdmann R. & Rottensteiner H. Pex7p and Pex20p of Neurospora crassa function together in PTS2-dependent protein import into peroxisomes. Mol. Biol. Cell 14, 810–821 (2003). 12589072 10.1091/mbc.E02-08-0539 PMC150010

[b28] Francisco T. . Ubiquitin in the peroxisomal protein import pathway. Biochimie 98, 29–35 (2014). 23954799 10.1016/j.biochi.2013.08.003

[b29] Hasan S., Platta H. W. & Erdmann R. Import of proteins into the peroxisomal matrix. Front. Physiol. 4, 261 (2013). 24069002 10.3389/fphys.2013.00261 PMC3781343

[b30] Hu J. . Plant peroxisomes: biogenesis and function. Plant Cell 24, 2279–2303 (2012). 22669882 10.1105/tpc.112.096586 PMC3406917

[b31] Liu X., Ma C. & Subramani S. Recent advances in peroxisomal matrix protein import. Cur. Opin. Cell Biol. 24, 484–489 (2012). 10.1016/j.ceb.2012.05.003 PMC3425728 22683191

[b32] Oeljeklaus S. . Identification of core components and transient interactors of the peroxisomal importomer by dual-track stable isotope labeling with amino acids in cell culture analysis. J. Proteome Res. 11, 2567–2580 (2012). 22375831 10.1021/pr3000333

[b33] Reguenga C., Oliveira M. E., Gouveia A. M., Sa-Miranda C. & Azevedo J. E. Characterization of the mammalian peroxisomal import machinery: Pex2p, Pex5p, Pex12p, and Pex14p are subunits of the same protein assembly. J. Biol. Chem. 276, 29935–29942 (2001). 11397814 10.1074/jbc.M104114200

[b34] Alencastre I. S. . Mapping the cargo protein membrane translocation step into the PEX5 cycling pathway. J. Biol. Chem. 284, 27243–27251 (2009). 19632994 10.1074/jbc.M109.032565 PMC2785652

[b35] Francisco T. . A cargo-centered perspective on the PEX5 receptor-mediated peroxisomal protein import pathway. J. Biol. Chem. 288, 29151–29159 (2013). 23963456 10.1074/jbc.M113.487140 PMC3790014

[b36] Gouveia A. M., Guimaraes C. P., Oliveira M. E., Sa-Miranda C. & Azevedo J. E. Insertion of Pex5p into the peroxisomal membrane is cargo protein-dependent. J. Biol. Chem. 278, 4389–4392 (2003). 12502712 10.1074/jbc.C200650200

[b37] Gouveia A. M. . Characterization of the peroxisomal cycling receptor, Pex5p, using a cell-free *in vitro* import system. J. Biol. Chem. 278, 226–232 (2003). 12411433 10.1074/jbc.M209498200

[b38] Gouveia A. M., Reguenga C., Oliveira M. E., Sa-Miranda C. & Azevedo J. E. Characterization of peroxisomal Pex5p from rat liver. Pex5p in the Pex5p-Pex14p membrane complex is a transmembrane protein. J. Biol. Chem. 275, 32444–32451 (2000). 10889202 10.1074/jbc.M004366200

[b39] Nair D. M., Purdue P. E. & Lazarow P. B. Pex7p translocates in and out of peroxisomes in Saccharomyces cerevisiae. J. Cell Biol. 167, 599–604 (2004). 15545321 10.1083/jcb.200407119 PMC2172567

[b40] Rodrigues T. A. . A PEX7-centered perspective on the peroxisomal targeting signal type 2-mediated protein import pathway. Mol. Cell. Biol. 34, 2917–2928 (2014). 24865970 10.1128/MCB.01727-13 PMC4135580

[b41] Oliveira M. E., Gouveia A. M., Pinto R. A., Sa-Miranda C. & Azevedo J. E. The energetics of Pex5p-mediated peroxisomal protein import. J. Biol. Chem. 278, 39483–39488 (2003). 12885776 10.1074/jbc.M305089200

[b42] Azevedo J. E., Costa-Rodrigues J., Guimaraes C. P., Oliveira M. E. & Sa-Miranda C. Protein translocation across the peroxisomal membrane. Cell Biochem. Biophys. 41, 451–468 (2004). 15509892 10.1385/CBB:41:3:451

[b43] Freitas M. O. . PEX5 protein binds monomeric catalase blocking its tetramerization and releases it upon binding the N-terminal domain of PEX14. J. Biol. Chem. 286, 40509–40519 (2011). 21976670 10.1074/jbc.M111.287201 PMC3220454

[b44] Lanyon-Hogg T., Hooper J., Gunn S., Warriner S. L. & Baker A. PEX14 binding to Arabidopsis PEX5 has differential effects on PTS1 and PTS2 cargo occupancy of the receptor. FEBS Lett. 588, 2223–2229 (2014). 24879895 10.1016/j.febslet.2014.05.038 PMC4065332

[b45] Neuhaus A. . A novel Pex14 protein-interacting site of human Pex5 is critical for matrix protein import into peroxisomes. J. Biol. Chem. 289, 437–448 (2014). 24235149 10.1074/jbc.M113.499707 PMC3879566

[b46] Carvalho A. F. . Ubiquitination of mammalian Pex5p, the peroxisomal import receptor. J. Biol. Chem. 282, 31267–31272 (2007). 17726030 10.1074/jbc.M706325200

[b47] Williams C., van den Berg M., Sprenger R. R. & Distel B. A conserved cysteine is essential for Pex4p-dependent ubiquitination of the peroxisomal import receptor Pex5p. J. Biol. Chem. 282, 22534–22543 (2007). 17550898 10.1074/jbc.M702038200

[b48] Fujiki Y., Miyata N., Matsumoto N. & Tamura S. Dynamic and functional assembly of the AAA peroxins, Pex1p and Pex6p, and their membrane receptor Pex26p involved in shuttling of the PTS1 receptor Pex5p in peroxisome biogenesis. Biochem. Soc. Trans. 36, 109–113 (2008). 18208396 10.1042/BST0360109

[b49] Platta H. W. . Ubiquitination of the peroxisomal import receptor Pex5p is required for its recycling. J. Cell Biol. 177, 197–204 (2007). 17452527 10.1083/jcb.200611012 PMC2064128

[b50] Debelyy M. O. . Ubp15p, a ubiquitin hydrolase associated with the peroxisomal export machinery. J. Biol. Chem. 286, 28223–28234 (2011). 21665945 10.1074/jbc.M111.238600 PMC3151067

[b51] Grou C. P. . Properties of the ubiquitin-pex5p thiol ester conjugate. J. Biol. Chem. 284, 10504–10513 (2009). 19208625 10.1074/jbc.M808978200 PMC2667737

[b52] Grou C. P. . Identification of ubiquitin-specific protease 9X (USP9X) as a deubiquitinase acting on ubiquitin-peroxin 5 (PEX5) thioester conjugate. J. Biol. Chem. 287, 12815–12827 (2012). 22371489 10.1074/jbc.M112.340158 PMC3339989

[b53] Mizuno Y. . Tysnd1 deficiency in mice interferes with the peroxisomal localization of PTS2 enzymes, causing lipid metabolic abnormalities and male infertility. PLoS Genet. 9, e1003286 (2013). 23459139 10.1371/journal.pgen.1003286 PMC3573110

[b54] Pan D., Nakatsu T. & Kato H. Crystal structure of peroxisomal targeting signal-2 bound to its receptor complex Pex7p-Pex21p. Nat. Struct. Mol. Biol. 20, 987–993 (2013). 23812376 10.1038/nsmb.2618

[b55] Madrid K. P., De Crescenzo G., Wang S. & Jardim A. Modulation of the Leishmania donovani peroxin 5 quaternary structure by peroxisomal targeting signal 1 ligands. Mol. Cell. Biol. 24, 7331–7344 (2004). 15314146 10.1128/MCB.24.17.7331-7344.2004 PMC506994

[b56] Kunze M. . Mechanistic insights into PTS2-mediated peroxisomal protein import: the co-receptor PEX5L drastically increases the interaction strength between the cargo protein and the receptor PEX7. J. Biol. Chem. 290, 4928–4940 (2015). 25538232 10.1074/jbc.M114.601575 PMC4335231

[b57] Croes K., Foulon V., Casteels M., Van Veldhoven P. P. & Mannaerts G. P. Phytanoyl-CoA hydroxylase: recognition of 3-methyl-branched acyl-coAs and requirement for GTP or ATP and Mg(2+) in addition to its known hydroxylation cofactors. J. Lip. Res. 41, 629–636 (2000). 10744784

[b58] Grou C. P. . Members of the E2D (UbcH5) family mediate the ubiquitination of the conserved cysteine of Pex5p, the peroxisomal import receptor. J. Biol. Chem. 283, 14190–14197 (2008). 18359941 10.1074/jbc.M800402200

[b59] Carvalho A. F. . The N-terminal half of the peroxisomal cycling receptor Pex5p is a natively unfolded domain. J. Mol. Biol. 356, 864–875 (2006). 16403517 10.1016/j.jmb.2005.12.002

[b60] Sullivan D. T. . Glyceraldehyde-3-phosphate dehydrogenase from Drosophila melanogaster. Identification of two isozymic forms encoded by separate genes. J. Biol. Chem. 260, 4345–4350 (1985). 2984203

[b61] Fujiki Y., Fowler S., Shio H., Hubbard A. L. & Lazarow P. B. Polypeptide and phospholipid composition of the membrane of rat liver peroxisomes: comparison with endoplasmic reticulum and mitochondrial membranes. J. Cell Biol. 93, 103–110 (1982). 7068748 10.1083/jcb.93.1.103 PMC2112093

[b62] Weibel E. R., Staubli W., Gnagi H. R. & Hess F. A. Correlated morphometric and biochemical studies on the liver cell. I. Morphometric model, stereologic methods, and normal morphometric data for rat liver. J. Cell Biol. 42, 68–91 (1969). 4891915 10.1083/jcb.42.1.68 PMC2107575

